# Genotype-Phenotype Insights of Inherited Cardiomyopathies—A Review

**DOI:** 10.3390/medicina60040543

**Published:** 2024-03-27

**Authors:** Oana Raluca Voinescu, Adina Ionac, Raluca Sosdean, Ioana Ionac, Luca Silvia Ana, Nilima Rajpal Kundnani, Stelian Morariu, Maria Puiu, Adela Chirita-Emandi

**Affiliations:** 1 Department of Cardiology, “Victor Babes” University of Medicine and Pharmacy, 300041 Timisoara, Romania; 2 Research Centre of Timisoara Institute of Cardiovascular Diseases, “Victor Babes” University of Medicine and Pharmacy, 300041 Timisoara, Romania; 3 Institute for Cardiovascular Diseases, Gheorghe Adam Street 13A, 300310 Timisoara, Romania; 4 General Medicine Faculty, “Vasile Goldis” West University, 473223 Arad, Romania; 5 Department of Microscopic Morphology, Genetics Discipline, Center of Genomic Medicine, University of Medicine and Pharmacy, “Victor Babeș” Eftimie Murgu Sq., 300041 Timisoara, Romania; 6 Regional Center of Medical Genetics Timiș, Clinical Emergency Hospital for Children “Louis Țurcanu”, Iosif Nemoianu Street N°2, 300011 Timisoara, Romania

**Keywords:** heritable cardiomyopathies, genetic testing, family screening, genetic counseling

## Abstract

*Background*: Cardiomyopathies (CMs) represent a heterogeneous group of primary myocardial diseases characterized by structural and functional abnormalities. They represent one of the leading causes of cardiac transplantations and cardiac death in young individuals. Clinically they vary from asymptomatic to symptomatic heart failure, with a high risk of sudden cardiac death due to malignant arrhythmias. With the increasing availability of genetic testing, a significant number of affected people are found to have an underlying genetic etiology. However, the awareness of the benefits of incorporating genetic test results into the care of these patients is relatively low. *Aim*: The focus of this review is to summarize the current basis of genetic CMs, including the most encountered genes associated with the main types of cardiomyopathies: hypertrophic, dilated, restrictive arrhythmogenic, and non-compaction. *Materials and Methods*: For this narrative review, we performed a search of multiple electronic databases, to select and evaluate relevant manuscripts. *Results*: Advances in genetic diagnosis led to better diagnosis precision and prognosis prediction, especially with regard to the risk of developing arrhythmias in certain subtypes of cardiomyopathies. *Conclusions*: Implementing the genomic information to benefit future patient care, better risk stratification and management, promises a better future for genotype-based treatment.

## 1. Introduction

**Figure 1:**
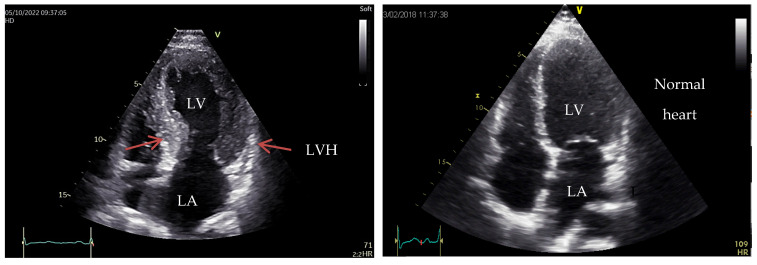
Two-dimensional echocardiography, apical four-chamber view showing important LV hypertrophy (LVH) and dilation of LA. Versus normal heart to the right side of the image.

**Figure 2:**
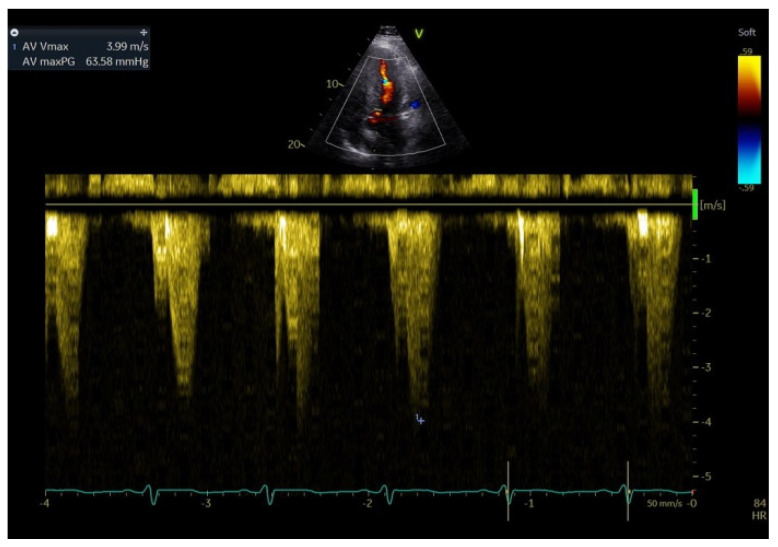
Continuous Wave Doppler in LV outflow tract measures a maximum gradient pressure of 63 mmHg.

**Figure 3:**
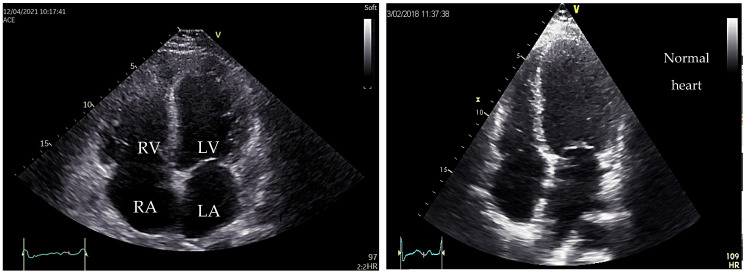
Two-dimensional echocardiography, apical four-chamber view showing biventricular dilation with spherical geometry and bilateral atrial enlargement, versus normal heart to the right side of the image.

**Figure 4:**
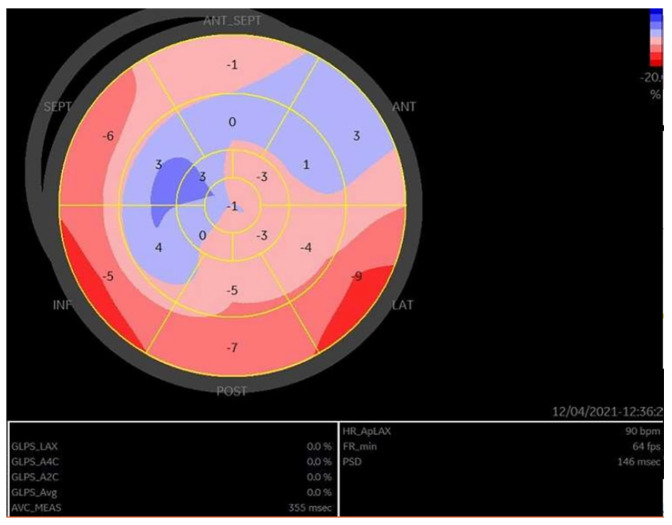
Speckle tracking emphasizes a left ventricle with reduced Global Longitudinal Strain (GLS) of −0%.

**Figure 5:**
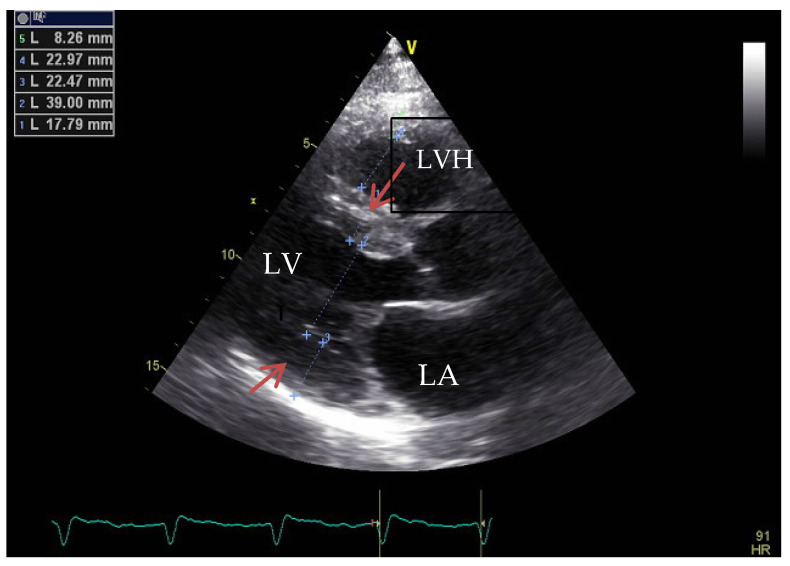
Two-dimensional echocardiography, parasternal long axis view showing an LV with concentric hypertrophy (LVH) and LA enlargement.

**Figure 6:**
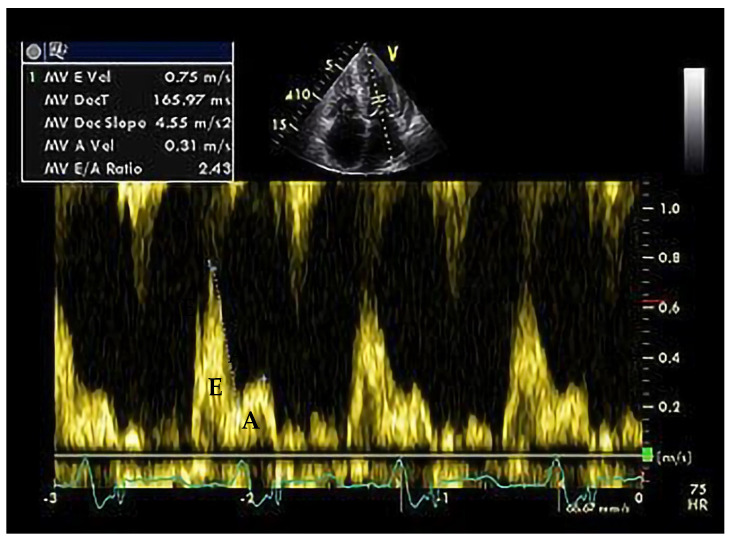
Pulsed Wave Doppler (PWD) at the mitral valve (MV) level, with E/A ratio of 2.4—suggestive for restrictive diastolic dysfunction.

**Figure 7:**
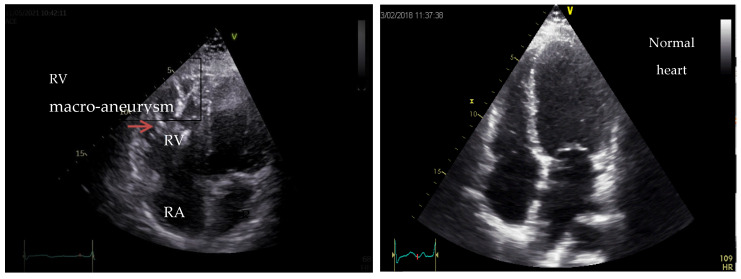
Two-dimensional echocardiography, apical four-chamber view showing right ventricular (RV) dilation with multiple macro-aneurysms and reduced systolic function versus normal heart to the right side of the image.

**Figure 8:**
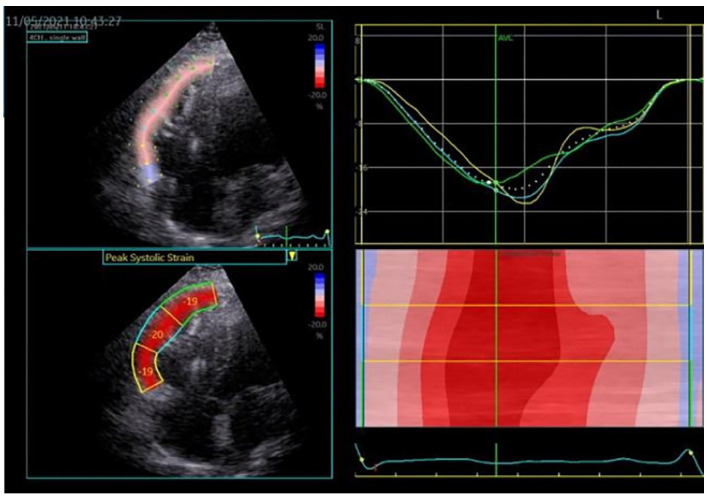
Speckle tracking analysis revealed reduced RV free wall longitudinal strain of −20% in a patient diagnosed with ACM predominantly affecting the RV.

Cardiomyopathies are defined as a group myocardial disorders in which the heart muscle becomes abnormal in structure and function leading to systolic and/or diastolic dysfunction and exhibits a higher risk of developing malignant arrhythmias [1]. The term cardiomyopathy was described for the first time in 1957, by Brigden, referring to patients with myocardial disease of unknown etiology, and some of them having familial aggregation [2]. The World Health Organization (WHO) and International Society and Federation of Cardiology (ISFC) presented the first classification of cardiomyopathies in 1980, based on the predominant structural and hemodynamic phenotype [3]. Five forms of the disease were formally classified at the beginning: hypertrophic cardiomyopathy (HCM; Figure 1 and Figure 2), dilated cardiomyopathy (DCM; Figure 3 and Figure 4), and restrictive cardiomyopathy (RCM; Figure 5 and Figure 6), arrhythmogenic ventricular cardiomyopathy (AVC; Figure 7 and Figure 8), and left ventricular non-compaction cardiomyopathy (LVNC). All cardiomyopathies initially were considered idiopathic, but later with the advancements in genetic testing they were divided into genetic/inherited forms and acquired/secondary forms. According to recently published guidelines regarding cardiomyopathies, a reclassification was established, which divides the cardiomyopathies as dilated cardiomyopathy, non-dilated cardiomyopathy, hypetrophic cardiomyopathy, restrictive cardiomyopathy and arrhythmogenic ventricular cardiomyopathy. A complex classification, “MOGES”, a comprehensive system which not only integrates the morphologic and functional characteristics but also includes the inheritance and genetic data including the: (M) from morpho-functional information, organ(s) involved (O), the genetic inheritance pattern (G), etiological annotation (E) and the functional status (S) of the patient, mostly based on heart failure symptoms [1,4].

Improvements in next-generation sequencing (NGS) technology for genetic clinical testing have provided access to larger gene panels, whole exome and whole genome sequencing over the years. Broader genetic testing increases the probability of identifying variants of incidental findings. All genetic testing can lead to discoveries of variants of uncertain significance (VUS) that represent a challenge for the clinical practice. Therefore, awareness of the benefits and hazards of genetic testing in order to choose the most suitable test, while also offering pretest genetic counselling, is valuable [5]. In the majority of cases, testing a small panel of specific genes in each type of cardiomyopathy offers good accuracy to detect single nucleotide substitutions for the identification of genetic defects such as: nonsense, missense or small deletion/insertion, while avoiding incidental discoveries. Specific cases, in which classic analyses turn out negative, might benefit from microarray tests in search of larger insertion/deletion variants > 25 nucleotides, but they represent < 1% of cases, as suggested by current studies, although these type of variants have been investigated less [4,5,6]. More recent advances in bioinformatics pipelines of NGS can provide information on copy number variants (CNV) from NGS [4,5].

Despite the rapid progress of molecular techniques over the past decades and that the increased genetic testing options have led to an improvement in understanding of the genetic complexity of these diseases, the actual global burden determined by genetic cardiomyopathies is still difficult to quantify, due to limited epidemiological studies [7]. In this review, we focus on the genetic background of the main primary cardiomyopathies, in order to improve medical management for the patients and their families.

## 2. Materials and Methods

We performed a search on the following electronic scientific resources: PubMed, Google Scholar, Web of Science, and Science Direct. Relevant open access articles employing the association between primary cardiomyopathies and genetic testing were identified. Key words used for the search included: “cardiomyopathy”, “genetic testing”, “next generation sequencing”, “molecular testing”, “genes”. We selected 76 articles, based on a database search published between 2004 and 2023. Manually, we checked the reference lists of the selected literature in order to validate the inclusion of genetic cardiomyopathies and the molecular characterization of these cardiac diseases based on the genetic testing.

### 2.1. Material Content

#### Basic Concepts of Clinical Genetics

Molecular genetics has shown that the classical Mendelian model of transmission might be more complex than previously thought. The model of inheritance should be determined in every case of genetic heart disease, which in daily busy clinical practice is difficult. Significant pitfalls need to be considered outside the Mendelian transmission. For example, diseases that were thought to be X linked recessive, for example Fabry disease, have shown that females may develop disease symptoms even with severe cardiac dysfunction, although the disease onset is later than for males. Moreover, an autosomal dominant cardiomyopathy may occur as an isolated case with a de novo variant [8]. The penetrance of a disease represents the proportion of variants carriers who develop the disease, irrespective of the presence of symptoms. For most autosomal recessive cardiac diseases (such as: arrhythmogenic ventricular cardiomyopathy), the penetrance is often complete. Penetrance in autosomal dominant cardiac diseases has proven to be rather age-related than incomplete, as illustrated by studies of molecular analyses. In hypertrophic cardiomyopathy the penetrance is estimated to be incomplete by the age of 30 (P = 50–80%) but nearly complete (P = 90–95%) by the age of 60 [9]. The majority of the monogenic cardiomyopathies tend to follow an autosomal dominant inheritance pattern, but cardiac manifestations can be very different, sometimes related to the age of onset. Variability of disease expression has been observed even between members of a family, which carry the same genetic variant. This has been attributed to intervention of additional influencing factors. Lifestyle, environmental factors, pharmacological agents, modifier genes, coexistence of different genetic variants may influence the phenotype of the disease [10]. Mitochondrial cardiomyopathies however represent a specific group of disorders determined by defects in mitochondrial DNA (mtDNA)/nuclear DNA (nDNA) resulting in mitochondrial function defects thus leading to the inability to produce adequate energy for the body, including the heart. The clinical picture of these diseases range from isolated cardiac dysfunction to cardiac involvement in the context of multisystem disorders such as neuromuscular and/or metabolic disease. Mitochondrial DNA dysfunction may lead to various cardiac phenotypes: including hypertrophic, dilated or non-compaction cardiomyopathy. Promising therapeutic approaches that target cell therapy, gene therapy, mitochondrial therapy, pharmacological therapy for a patient with mitochondrial cardiomyopathy are emerging fast at the preclinical level but clinical translation is still lacking [11].

The ClinGen Gene Curation Working Group built a method to qualitatively define the “clinical validity” of the genes-disease relationship by creating a classification method based on the strength of evidence that supports this relationship. A gene which is interpreted as “definitive” in relation to a particular disease has been repeatedly demonstrated in both research and clinical settings and has been reported in at least two independent scored publications documenting human genetic evidence over time. Gene-disease relation with “strong” evidence is supported by numerous unrelated probands identified with variants with multiple evidence favoring disease causality. Gene-disease pair with “moderate” evidence imply some convincing genetic evidence and no data that contradicts the role of the gene in the noted disease has emerged. The “limited” association is considered when the variants have some support for pathogenic impact, but there is little to no functional evidence to prove it. When existing genetic evidence has been ruled out, leaving the gene with no valid evidence remaining after an initial claim, leads to a “refuted” association [12].

## 3. Genetic Testing? Whom, When and How

A methodical approach to the genetic diagnosis of a heritable cardiomyopathy should consist in obtaining a detailed history of the disease, physical examination with focus on syndromic cardiovascular disease and a complete family history incorporating a three-generation pedigree [6]. Genetic testing should be integrated in the work-out of any primary cardiomyopathy. It should be initiated with the patient having symptoms in the family or early onset of the disease, as this increases the probability of finding a genetic cause. There are no clear recommendations regarding the best timing for performing the genetic testing [11]. It is recommended to be performed at the moment of cardiomyopathy diagnosis because genetic results may guide the management [12]. The complexity of gene panels have improved over time, especially those for DCM [13]. For patients with negative or uncertain results at previous tests, reinterpretation of raw sequencing data or retesting may be an option. This indication is based on the fact that larger datasets become available with advanced research and gene variants can be reclassified over time [14,15]. This means that VUS can become likely pathogenic, or variants previously considered likely pathogenic can be downgraded to VUS. Next-generation sequencing using a multi-gene panel is the best option for these diseases with heterogeneous genetic background, as they are technically feasible and cost-efficient [16]. These large gene panels have the advantage of increasing the odds of identifying a genetic etiology, especially in patients with uncertain phenotypes or without pathognomonic manifestations. With larger gene panels, the likelihood of identifying a variant of uncertain significance is higher and the overlap of genes responsible for different types of cardiomyopathies makes the interpretation harder. Family testing could be supported for reclassification of a VUS variant, if identified in multiple members of the family with a similar phenotype, or on the contrary if identified as de novo, from healthy parents and siblings. It is important that the ordering physician has a good knowledge of the advantages but also the limitations of specific test types in order to select the most appropriate test for their patient [17]. Once a pathogenic or likely pathogenic variant has been identified in the proband, Sanger sequencing and targeted gene analysis should be performed for the other family members. The rationale behind identifying family genetic risk is that those found to be at-risk can undergo periodic evaluation/systematic screening to detect the earliest manifestations of the disease. Thus, in such situations, the first symptom or imagistic sign would prompt early management, including lifestyle changes, cardio-protective treatment to slow down disease progression, and devices implantation for reducing the risk of sudden cardiac death. Identifying healthy carriers of the pathological gene may also have implications regarding family planning and reproductive decisions [18]. Combined medical education and genetic counseling provided by the cardiologist and genetic physician is crucial for achieving the best medical management.

## 4. Hypertrophic Cardiomyopathy

Hypertrophic cardiomyopathy (HCM) is defined by the presence of inappropriate left ventricular hypertrophy unexplained by any cardiac or systemic abnormal loading condition. The diagnostic criteria is defined by maximum left ventricular wall thickness of ≥15 mm. For first-degree relatives, unexplained wall thickness of ≥13 mm in any segment is suggestive for the diagnosis [19]. It is the most frequently encountered inherited cardiomyopathy, with a prevalence in the general population of 1:500, or even higher [20,21]. 

As an example, we present in Figure 1 and Figure 2, the images of the heart in a patient diagnosed with HOCM, carrier of a pathogenic gene variant in the *MYH7* gene (Courtesy: Prof. Dr. Adina Ionac-Institute of Cardiovascular Disease, Timisoara, Romania). 

There are several phenotypes described. The symmetrical form of HCM accounts for 33% of cases having concentric thickening of the left ventricle with a small ventricular cavity diameter. The obstructive form of HCM (HOCM) involves predominant thickening of the basal septum, narrowing the left ventricular outflow tract. Anterior displacement of the papillary muscles is common and anterior mitral leaflet systolic motion (SAM) into the left ventricle outflow tract obstruction (LVOT) during systole is responsible for LVOT (Figure 2). This pattern has been considered in past as pathognomonic for HCM, but it has been described also in non-sarcomeric HCM. HCM may involve any segment of the left ventricle and there have been variants described that involve mid-ventricular or apical LV hypertrophy.

Symptoms may vary a great deal, related to the severity of hypertrophy and grade of fibrosis. Patients may be asymptomatic or complain about heart failure symptoms due to diastolic dysfunction. Angina is common and can be explained by microvascular dysfunction and relative myocardial ischemia. Narrowing of the small intramural coronary arteries due to wall media thickening by muscle cell hyperplasia may lead to fibrosis and the development of the systolic dysfunction in advanced stages. Furthermore, myocardial fibrosis has been correlated to the risk of sudden cardiac death through malignant arrhythmias. Patients with asymmetric hypertrophy in particular have the risk of developing syncope during physical exertion due to dynamic LVOT obstruction [22].

HCM is an autosomal dominant disease in most of cases, commonly caused by variants in genes encoding sarcomere proteins [20,23]. In patients fulfilling diagnostic criteria for HCM, genetic testing is positive in 30–60% of cases and even higher in cases of familial aggregation. About 70% of these variants are localized in sarcomere genes encoding cardiac β-myosin heavy chain (*MYH7*) and cardiac myosin binding protein C (*MYBPC3*) [24]. It is estimated that 50–60% of patients who have a family member with HCM carry a pathogenic gene variant [25,26]. To date, there have been multiple gene variants responsible for HCM described [Table 1]. The most frequent genes involved are *MYBPC3*, *MYH7*, *TNNT2*, *TNNI3*, *ACTN2*, and *TPM1*. Different variants of the same gene have been related to different grades of disease severity and prognosis. For example, pathogenic variants in the cardiac β-myosin heavy chain gene are related to severe forms of HCM with onset at early age and high risk of sudden cardiac death. Disease-causing variants in Troponin T despite inducing only mild hypertrophy are associated with poor outcomes and high risk of malignant arrhythmias. On the other hand, myosin-binding protein C variants are associated with late clinical onset and a more benign clinical course. Expression variability is emphasized by different clinical response and evolution between members of the same family carriers of an identical genetic variant. In a study published in 2019 by Maurizi et al., it was stated that only 10% of the asymptomatic HCM-carriers of pathogenic variants developed cardiac disease during a clinical follow-up of 6 ± 2 years [27]. According to 2104 ESC Guidelines for diagnosis and management of HCM, genetic testing is recommended in patients who fulfill diagnostic criteria to confirm the diagnosis (indication class I, level B), especially where familial aggregation is present [19,28]. 

Etiological diagnosis is challenging when HCM is represented by multiple phenocopies. These include physiological transitory changes found in athletes, metabolic and other hereditary diseases such as Fabry disease or cardiac amyloidosis. A correct diagnosis is crucial since these diseases have a different evolution and benefit from specific treatment further influencing the prognosis [29]. For example, in patients with sarcomeric hypertrophic obstructive cardiomyopathy, mavacamten treatment, an inhibitor a cardiac myosin ATPase, was found to be associated with favorable remodeling of myocardium and improvement in functional capacity of these patients (EXPLORER HCM Trial) [30]. Invasive treatment to reduce gradient form the left ventricle outflow tract may be considered in patients with a maximum gradient ≥ 50 mmHg, with heart failure severe symptoms and syncope despite maximum tolerated medical therapy. This therapeutic approach include alcohol septal ablation or surgical procedure for ventricular septal myectomy. Patients with HCM express an annual incidence for death of 1–2%, SCD, heart failure, and thrombo-embolism events being the major causes of death. Calculation of SCD risk score is indicated in these patients and ICD implantation in primary prevention of SCD in recommended according to the score value [4].

**Table 1:** List of common genes and patterns of inheritance in cardiomyopathies (alphabetical), curated by ClinGen [31] and literature review.

Gene Symbol	Disease OMIM #	Gene Name	Mode of Inheritance	HCM	DCM	RCM	ACM	LVNC
*ABCC9*	601439	ATP-binding cassette	AD	-	Limited	-	-	-
*ACTC1*	102540	Actin alpha	AD	Definitive	Moderate	-	-	-
*ACTN2*	102573	Actinin alpha 2	AD	Definitive	Definitive	Definitive	Definitive	Definitive
*ALMS1*	606844	Centrosome and basal body associated protein	AR	-	Definitive			
*ALPK3*	617608	Alpha kinase 3	AR	Definitive	-	-	-	-
*ANKRD1*	609599	Ankyrin repeat domain-containing protein 1	AD	Disputed	Limited	-	-	-
*ARVD3*	602086	Arrhythmogenic right ventricular dysplasia, familial, 3	AD	-	-	-	Limited evidence	-
*ARVD4*	602087	Arrhythmogenic right ventricular dysplasia, familial, 4	AD	-	-	-	Limited evidence	-
*ARVD6*	604401	Arrhythmogenic right ventricular dysplasia, familial, 6	AD	-	-	-	Limited evidence	-
*BAG3*	603883	Bcl2-associated athanogene 3	AD	-	Definitive	-	-	-
*BRAF*	164757	B-Raf proto-oncogene serine/threonine kinase	AD	Definitive	-	-	-	-
*CASQ2*	114251	Calsequestrin 2	AR, AD	Disputed	-	-	-	-
*CAV3*	601253	Caveolin 3	AD	Definitive	-	-	-	-
*CRYAB*	123590	Crystallin	AD	Limited evidence	Limited evidence	-	-	-
*CSRP3*	600824	Cysteine and glycine rich protein 3	AD	Definitive	Limited			
*CTF1*	600435	Cardiotrophin 1	AR, AD	-	Limited			
*CTNNA3*	607667	Catenin alpha 3	AD	-	-	-	Limited	-
*DES*	125660	Desmin	AD, AR	-	Definitive	-	Moderate	-
*DMD*	300377	Dystrophin	XLR	-	Definitive * Duchenne muscular dystrophy Becker muscular dystrophy	-	-	-
*DOLK*	610746	Dolichol kinase	AR	-	Limited	-	-	-
*DSC2*	600271	Desmocollin 2	AD, AR	-	Limited	-	Definitive	-
*DSG2*	125671	Desmoglein 2	AD	-	Limited	-	Definitive	-
*DSP*	125485	Desmoplakin	AD, AR	Disputed	Definitive	-	Definitive-	-
*DTNA*	601239	Dystrobrevin, alpha	AD	-	Limited	-	-	Limited Evidence
*EMD*	300384	Emerin	XLR1	- Definitive * Emery-Dreifuss Muscular Dystrophy	Definitive * Emery-Dreifuss Muscular Dystrophy	-	-	-
*EYA4*	603550	Eyes absent Drosophila homolog 4	AD	-	Limited			
*FHL1*	300163	Four and a half LIM domain 1	XL	Definitive * Emery-Dreifuss Muscular Dystrophy and related phenotypes	Definitive * Emery-Dreifuss Muscular Dystrophy and related phenotypes	-	-	-
*FKRP*	606596	Fukutin-related protein	AR	-	Definitive * Limb Girdle Muscular Dystrophy			
*FKTN*	607440	Fukutin	AR	-	Definitive * Limb Girdle Muscular Dystrophy			
*FLNC*	102565	Filamin C	AD	Definitive	Definitive	Limited evidence	Limited evidence	-
*GAA*	606800	Glucosidase alpha	AR	Definitive	-	-	-	-
*GATA4*	600576	Gata-binding protein 4	AD	-	Limited	-	-	-
*GATAD1*	614518	Gata zinc finger domain-containing protein 1	AR	-	Limited	-	-	-
*GLA*	300644	Galactosidase alpha	XL	-	-	Definitive	-	-
*HCN4*	605206	Hyperpolarization-Activated. Cyclic nucleotide-gated. Potassium Channel 4	AD	-	-	-	-	Limited
*HRAS*	190020	HRas proto-oncogene, GTPase	AD	Definitive	-	-	-	-
*ILK*	602366	Integrin-linked kinase	AD	-	Limited	-	-	-
*JPH2*	605267	Junctophilin 2	AD	Moderate	Moderate	-	-	-
*JUP*	173325	Junction plakoglobin	AD, AR	-	-	-	-	Definitive * Naxos Disease
*LAMA4*	600133	Laminin alpha-4	AD	-	Limited			
*LAMP2*	309060	Lysosome-associated membrane protein 2	XLD	Definitive	Limited			
*LDB3*	605906	LJm domain-binding 3	AD	Limited evidence	Limited		Disputed	Limited evidence
*LMNA*	150330	Lamin A/C	AD, AR	Limited evidence	Definitive		Limited	Limited evidence
*MAP2K1*	176872	Mitogen activated protein kinase 1	AD	Definitive * Cardiofaciocutaneous syndrome	-	-	-	-
*MAP2K2*	601263	Mitogen activated protein kinase 2	AD	Definitive * Cardiofaciocutaneous syndrome	-	-	-	-
*MYBPC3*	600958	Myosin-binding protein C, cardiac	AD	Definitive	Limited	-	Limited	Limited evidence
*MYH6*	160710	Myosin heavy chain 6	AD	Limited	Limited	-	-	-
*MYH7*	160760	Myosin, heavy chain 7, cardiac muscle, beta	AD	Definitive	Definitive	Limited evidence	Limited	Limited evidence
*MYL2*	160781	Myosin light chain 2	AD	Definitive	Limited	-	-	-
*MYL3*	160790	Myosin light chain 3	AD, AR	Definitive	Disputed	Limited evidence	Limited	-
*MYLK2*	606566	Myosin light chain kinase 2	AD	Disputed	Limited	-	-	-
*MYOM1*	603508	Myomesin 1	AD	Disputed	-	-	-	-
*MYOT*	604103	Myotilin	AD	-	Limited	-	-	-
*MYOZ2*	605602	Myozenin 2	AD	Disputed	Limited	Limited evidence	-	-
*MYPN*	608517	Myopalladin	AD	Disputed	Limited	Limited evidence	-	-
*NEBL*	605491	Nebulette	AD	-	Limited	-	-	-
*NEXN*	613121	Nexilin	AD	Limited	Moderate	-	-	-
*NKX2-5*	600584	Nk2 homeobox 5	AD	-	Limited	-	-	-
*NRAS*	164790	Neuroblastoma Ras viral oncogene homolog	AD	Definitive * Noonan syndrome	-	-	-	-
*PDLIM3*	605899	Pdz and lim domain protein 3	AD	Limited	Disputed	-	-	-
*PKP2*	602861	Plakophilin 2	AD	-	Disputed	-	Definitive	-
*PLN*	172405	Phospholamban	AD	Definitive	-	-	Moderate	-
*PRDM16*	605557	Pr domain-containing protein 16	AD	-	Limited-	-	-	Limited
*PRKAG2*	602743	Protein kinase amp-activated non-catalytic	AD	Definitive	Limited evidence	-	-	-
*PTPN11*	176876	Protein tyrosine phosphatase non-receptor type 11	AD	Definitive * Noonan Syndrome	-	-	-	-
*RAF1*	164760	V-Raf-1 murine leukemia viral oncogene homolog 1	AD	Definitive * Noonan Syndrome	-	-	-	-
*RBM20*	613171	RNA-binding motif protein 20	AD	Limited	Definitive	-	-	-
*RIT1*	609591	Ras-like without Caax 1	AD	Definitive * Noonan Syndrome	-	-	-	-
*SCN5A*	600163	Sodium channel, voltage-gated, type V, alpha subunit	AD	-	Definitive	-	Limited	-
*SGCA*	600119	Sarcoglycan alpha	AR	-	Definitive * Limb Girdle Muscular Dystrophy	-	-	-
*SGCB*	600900	Sarcoglycan beta	AR	-	Definitive * Limb Girdle Muscular Dystrophy	-	-	-
*SGCD*	601411	Sarcoglycan delta	AD, AR	-	Limited Definitive * Limb Girdle Muscular Dystrophy	-	-	-
*SHOC2*	602775	Soc-2 homolog	AD	Definitive * Noonan Syndrome	-	-	-	-
*SLC25A4*	103220	Solute carrier family 25	AD, AR	Definitive	Limited * Leigh Syndrome			
*SOS1*	182530	Son of sevenless Drosophila homolog 1	AD	Definitive * Noonan Syndrome	-	-	-	-
*TAZ*	300394	Tafazzin	XL R	-	Definitive * Barth Syndrome	-	-	Limited Evidence
*TBX20*	606061	T-box 20	AD	-	Limited	-	-	Limited Evidence
*TCAP*	604488	Titin-Cap	AR	Disputed	Limited	-	-	-
*TGFB3*	190230	Transforming growth factor beta 3	AD	-	-	-	Limited	-
*TMEM43*	612048	Transmembrane protein 43	AD	-	-	-	Definitive	-
*TNNI3*	191044	Troponin I type 3 (cardiac)	AD	Definitive	Moderate	Limited evidence	-	-
*TNNC1*	191040	Troponin C type 1	AD	Definitive	Definitive	-	-	-
*TNNT2*	191045	Troponin T type 2 (cardiac)	AD	Definitive	Definitive	Limited evidence	-	Limited evidence
*TOR1AIP1*	614512	Torsin-1a-interacting protein 1	AR	-	Limited evidence	-	-	-
*TPM1*	191010	Tropomyosin 1 (alpha)	AD	Definitive	Moderate	Limited evidence	-	-
*TTN*	188840	Titin	AD, AR	Limited	Definitive	-	Limited	-
*TTR*	176300	Transthyretin	AD	Definitive	-	Definitive	-	-
*TXNRD2*	606448	Thioredoxin reductase 2	AD, AR	-	Limited evidence	-	-	-
*VCL*	193065	Vinculin	AD	Disputed	Moderate	-	-	Limited evidence

* Syndromic cardiomyopathy, ACM: arrhythmogenic cardiomyopathy, AD: autosomal dominant, AR: autosomal recessive, XL: X linked, HCM: hypertrophic cardiomyopathy, DCM: dilated cardiomyopathy, RCM: restrictive cardiomyopathy, LVNC: left ventricular non-compaction.

## 5. Dilated Cardiomyopathy (DCM)

Dilated cardiomyopathy is a disease of the heart muscle characterized by ventricular dilatation and systolic dysfunction, in the absence of abnormal loading conditions such as: coronary artery disease, significant valvulopathies, or viral infections such as COVID-19 [30,32]. The clinical picture is represented by heart failure (HF), arrhythmias, conduction system disorders or SCD. Globally, DCM represents the main cause of HF, reaching almost half of the patients in HF registries and represents a leading cause of heart transplant [33]. 

The prevalence of DCM has varied a lot over time and it is difficult to establish; recent studies have reached to a prevalence 1:400 [17]. About 30–50% of cases have positive family history, with autosomal dominant pattern of transmission but with variable penetrance. Autosomal recessive, X-linked, and mitochondrial inheritance patterns have been described but they are more frequently diagnosed during childhood [1,34,35]. DCM has been classified into non-syndromic forms, when the defect is localized only to the heart, and syndromic forms which involves systemic disease manifestations. 

As an example, we present in Figure 3 and Figure 4, the images of the heart in a patient diagnosed with dilated cardiomyopathy, carrier of the pathogenic variant in the *TTN* gene (Courtesy: Dr Raluca Sosdean).

According to Human Gene Mutation Database and the Online Mendelian Inheritance in Man [36], initially more than 100 genes have been linked to DCM, some of them being refuted after advanced data analysis. They are involved in coding proteins of the different cell structures such as: cytoskeleton, sarcomere, nuclear membrane and ion channels, with an important overlap with other types of cardiomyopathies [37]. The majority of disease-causing variants are specific to a family and about 10% of patients seem to have at least two variants involved [35,38]. Non-syndromic DCM are most common determined by truncating variants of the Titin gene (*TTN*) [39,40]. Associated neuromuscular symptoms in the patient or relatives is an important criterion for gene analysis since variants in *LMNA* are involved in DCM associated with neuromuscular disease (limb girdle muscular dystrophy). Laminopathies are frequently associated with prolonged PR interval on the ECG, indicator of cardiac conduction disorder [41,42]. 

Recent data indicates that sarcomere genes *MYH7*, *TNNT2* and *TPM1*, usually involved in HCM etiology, represent 2–4% of the variants identified in DCM, while *MYBPC3* variants are rare [43]. Phospholamban (*PLN*) variants, which encodes a transmembrane protein that inhibits sarcoplasmic reticulum Ca^2+^-ATPase, have been associated with DCM. Variable phenotypes have been described, with different phenotypes from mild cases to early onset disease associated with lethal ventricular arrhythmias [44,45]. In centers with cardiovascular genetic expertise, the rate of genetic diagnosis in non-syndromic DCM is about 46–73% [46]. Acquiring more information about the genotype–phenotype associations could improve the specificity of genetic testing and lead to cost efficient analysis.

HF medication has proved to have benefic effect on LV remodeling in symptomatic patients that associate LV dysfunction, first-line heart failure therapy: angiotensin converting enzyme inhibitors, angiotensin receptor blockers, beta-blockers, diuretics, and aldosterone antagonists should be considered in these patients to prevent LV dilatation and dysfunction progression. An ICD is recommended in DCM patients with purpose of increasing the survival; arrhythmic risk calculators may be useful tools to predict the risk of SCD, where available. Progression towards end-staged heart failure despite maximally tolerated drug therapy or intractable ventricular arrhythmia leads to indication of cardiac transplantation [4].

## 6. Non-Dilated Cardiomyopathy

A phenomenon characterized by intermediate phenotypes that do not meet standard disease criteria despite the identification of myocardial disease on cardiac imaging or tissue evaluation has been described [46]. Therefore, 2023 ESC Guidelines for the management of cardiomyopathies proposed the introduction of a new term “non-dilated left ventricular cardiomyopathy” (NDLVC). This phenotype defined by the existence of non-ischemic LV scar or fatty tissue replacement on MRI scans, without cardiac chamber dilation, regardless of the presence of global or regional contractility dysfunction. Isolated global LV hypokinesia without fibrosis is also included in this category. The NDLVC phenotype includes patients that previously been considered as having DCM without LV dilatation, left dominant arrhythmogenic left ventricular cardiomyopathy but often without having complete diagnostic criteria for ARVC. The diagnosis of an NDLVC phenotype should initiate a multi-variable approach including clinical examination, family history, cardiac imaging, myocardial tissue characterization, Holter ECG monitoring for arrhythmic burden and genetic testing. These entire tests can lead to a specific etiological diagnosis with implications for medical/interventional treatment and follow-up [4].

## 7. Restrictive Cardiomyopathy (RCM)

Restrictive cardiomyopathy is characterized by diastolic dysfunction of a non-dilated LV with normal systolic function. The clinical presentation is heterogeneous and it is correlated with high cardiac filling pressures due to a non-compliant left ventricle [46]. Patients may present signs and symptoms of heart failure, tachyarrhythmia’s, or sudden cardiac death. Echocardiography together with electrocardiography is the most important tool for the diagnosis, revealing normal ventricular volumes with normal/mild hypertrophic walls, restrictive diastolic dysfunction and bi-atrial dilation and hence and also of their arrhythmic complications including atrial fibrillation and ventricular tachycardia, that can be complicated by sudden death or stoke [47]. These changes can be augmented by the association of other cardiovascular risk factors such as obesity, dyslipidemia, diabetes isolated or grouped as in the diagnosis of metabolic syndrome [48,49].

As an example, we present in Figure 5 and Figure 6, the echocardiographic images of a patient diagnosed with restrictive cardiomyopathy secondary to cardiac amyloidosis, carrier of a pathogenic variant in the transtiretin (*TTR*) gene (Courtesy: Prof. Dr. Adina Ionac).

RCM is mainly classified as primary and secondary. The primary RCM are histologically characterized by interstitial fibrosis and include the endomyocardial fibrosis (EMF), Loeffler’s endocarditis, and idiopathic forms. Secondary forms of RCM are more common and are subclassified as infiltrative (amyloidosis, sarcoidosis) or as storage disorders (haemochromatosis, glycogenosis, Fabry disease) and non-infiltrative (anthracycline induced toxicity carcinoid syndrome) [50]. The most frequent genetic variants found in RCM cases are in sarcomeric genes, such as *TNNI3* (most common), *TNNT2*, *MYH7*, *ACTC1*, *TPM1*, *MYL3*, and *MYL2* also known to cause HCM [51,52]. Therefore, genetic testing for RCM should include HCM genes [53]. Most variants are localized in genes encoding sarcomeric proteins, some in sarcomere-associated proteins like small heatshock protein: crystallin αB, or their binding partners *LBAG3*—proteins whose dysfunction potentially leads to the accumulation of aggregated proteins. There have also been variants described in genes whose proteins are not directly involved in contractile function such as: desmin, filamin C and crystallin αB [54,55]. Desmin variants have been usually associated with DCM, however, a p.*E413K* variant has been described in a family with a history of SCD, in which three family members were diagnosed with RCM [56]. Familial aggregation is identified in up to 30% of cases in RCM. 

Infiltrative causes of RCM are mainly represented by cardiac amyloidosis—a disorder characterized by deposition of insoluble fibrillar protein-like filaments between the muscle fibers, small arteries media, and peripheral nervous system. It is classifies into two main types: light chain (AL) and the transthyretin amyloidosis (ATTR). The latter type includes a hereditary sub-type caused by variants of the transthyretin protein (genetic), and a more common wild-type ATTR (ATTRwt) which is age-related (senile) [57]. If ATTR cardiomyopathy is identified, then genetic sequencing of the *TTR* gene is necessary. Differentiating ATTRv from ATTRwt is critical because confirmation of ATTRv should trigger genetic counseling and potential screening of family members. Furthermore, identification of Val122Ile variant involves a more aggressive progression with median survival after diagnosis in untreated patients of 2.5 years [58]. Hemochromatosis is a disorder which involves abnormal systemic deposition of iron. This disease has been associated with low-penetrance autosomal dominance of pathogenic variants identified in the *HFE* gene [59]. A pathogenic variant of *BTNL2* gene has been associated with sarcoidosis, a granulomatous systemic disease characterized by restrictive cardiomyopathy, pulmonary and skin infiltration [60]. The importance of a correct etiological diagnosis the disease benefit from specific treatment. According to the results of ATTR-ACT randomized trial (Safety and Efficacy of Tafamidis in Patients wth Transthyretin Cardiomyopathy), Tafamidis treatment was associated with a significantly lower mortality and cardiovascular hospitalization [61].

## 8. Arrhythmogenic Cardiomyopathy (ACM)

Arrhythmogenic ventricular cardiomyopathy (AVC) is characterized by the progressive loss of myocytes due to the replacement of myocardium by fibro-adipose tissue. Groups of myocytes are surrounded by fatty-fibrous tissue creating an electrical pathway for malignant ventricular arrhythmias [62]. Sudden cardiac death is more frequent in young individuals and is commonly precipitated by exercise. Though it was firstly thought to be a disease isolated to the right ventricle, now it is known that it can affect both ventricles or predominantly LV. Subsequently, the initially name of right ventricular dysplasia was replaced by “arrhythmogenic cardiomyopathy”.

As an example, we present in Figure 7 and Figure 8 the images of the heart in a patient with right ventricle arrhythmogenic cardiomyopathy, a carrier of a pathogenic variant in the *PLN* gene. (Courtesy: Dr Raluca Sosdean, Institute of Cardiovascular Disease Timisoara).

In 2020, Padua criteria for positive diagnosis were proposed, focused on the most affected ventricle. They include morpho-functional changes, tissue characterization, ECG disorders, ventricular arrhythmias and family history/genetic variants [63]. Up to 50% of AVC cases have positive family history [46]. AVC frequently has an autosomal dominant transmission with incomplete penetrance, although two autosomal recessive forms have been described (Naxos disease and Carvajal syndrome) [64]. ACM has a heterogeneous genetic background, mainly involving variants in genes encoding structural proteins as desmosome proteins: plakoglobin, desmoplakin, plakophillin, accounting for up to 60% of affected patients [65]. Variants in *PLN* encoding phospholamban have been identified to cause 10–15% of all ACM patients in Netherlands. Loss of desmosomal integrity affect myocytes gap junctions and electrical propagation leading to ventricular arrhythmias in the absence of extensive structural changes. Other ACM target genes encode the cardiac sodium channel, titin, lamin A/C transmembrane protein 43, and filamin C [66]. Overall, the most commonly mutated gene is plakophilin, which accounted for 46–61% of patients from two registries [67].

To control arrhythmia-related symptoms in patients with ARVC, beta-blockers represent the first option of treatment by reduction of adrenergic tone, particularly during exercise. Amiodarone is often used when other beta-blockers fail to control arrhythmias. Sotalol has been used for many years, but data regarding the efficacy is limited or conflicting. Flecainide should be considered when single-agent treatment has failed. Experience with other antiarrhythmic medications is limited to very small case series. A proportion of patients with high arrhythmic burden require invasive procedures for ablation useful in reducing the risk of electrical storm. Patients with increased risk of SCD will require ICD implantation. Discontinuation of intense physical exercise has proven to be effective against disease progression and reducing the ventricular arrhythmia burden [4].

## 9. Left Ventricular Non-Compaction

Left ventricular non-compaction is morphologically characterized by a thinned, compact myocardial layer and a thickened trabecular myocardial layer composed of excessive trabeculations and deep recesses [68]. Abnormal intrauterine myocardial compaction occurs in the final phase of myocardial morphogenesis, during the first trimester of pregnancy [69]. Imagistic diagnostic criteria are based on echocardiography or cardiac magnetic resonance imaging. The most widely used CMR indicator in clinical studies is a non-compacted-to-compacted (NC/C) ratio > 2.3, according to criteria established by Petersen et al. associated with LV dysfunction and dilation [70,71]. LVNC primarily involves the LV, although cases of biventricular or isolated RV non-compaction have been described [72]. Clinical manifestations in patients with LVNC vary from asymptomatic patients to congestive heart failure symptoms, arrhythmias, thromboembolic complications, or sudden cardiac death. Diagnostic testing in patients with LVNC appear to have a detection rate of clinically significant variants in 35–40% of individuals, with sarcomere-encoding genes most commonly found to be mutated [73]. Physiologic hyper trabeculation may be present also in individuals without a cardiomyopathy with increased incidence in certain situations, such as pregnancy and intense physical exercise [74,75]. For this reason, recently it has been proposed to replace the term of LVNC cardiomyopathy by hyper-trabeculation. The Task Force does not consider LVNC to be a specific type of cardiomyopathy but a phenotypic feature that can be associated with other structural abnormalities, such as: ventricular hypertrophy, dilatation, and/or systolic impairment. The term ‘hypertrabeculation’ if preferred for this phenotype, rather than LVNC, as recommended by the Guideline of Cardiomyopathies published in August 2023 [4], due to the fact that this structural feature can be transitory or developed during adulthood. LVNC may be associated with congenital heart disease (CHD), including septal defects (ASD, VSD), hypoplastic left heart syndrome (HLHS), pulmonic stenosis (PS) and Ebstein’s disease [71]. Variants in sarcomere-encoding genes (*MYH7*, *ACTC*, *TNNT2*, *MYBPC3*, *TMP1*, and *TNNI3*) and the Z-line protein-encoding *ZASP/LDB3* gene account for 20% or more of isolated LVNC, especially diagnosed during childhood [76]. Other variants described in the development of LVNC are affecting ion channels (*SCN5A*, *HCN4*, and *RYR2*) and mitochondria (*NNT*, *TAZ*). Pathogenic variants of the *SCN5A*, *LMNA*, *RBM20*, *TTN*, and *DES* genes have been associated with LVNC and increased risk of arrhythmias [77]. 

## 10. Conclusions

Inherited cardiomyopathies represent a heterogeneous group of genetic myocardial diseases with high risk of morbidity and mortality. In recent years, the number of patients diagnosed with hereditary CMs has grown, due to increased awareness and advances in cardiac imaging modalities. Next-generation sequencing methods have revolutionized genetic testing, providing identification of genetic cause of inherited cardiac diseases. Increasing numbers of rare genetic variants have been validated, new genetic markers for cardiomyopathy risk have been identified and a new pathway for future personalized care in cardiomyopathies is now available. However, diagnostic precision and potential therapeutic implications of genetic testing are still limited due to wide genetic heterogeneity, new identified variants and multiple genetic variants association. Influencing factors such as: incomplete penetrance, variable expression, impact of modifier genes, the environmental factors and furthermore the incomplete understanding of VUS, still represent significant challenges. Studying early manifestations of CMs can help identify genotype-phenotype correlations and potentially find disease-modifying treatments. Pleiotropy is a crucial aspect of genotype-phenotype relationships in the context of cardiomyopathies. Single genetic variants may contribute to the development of different forms of cardiomyopathies or even affect non-cardiac phenotypes [78,79]. Integrating genetic testing into diagnostic calculation of inheritable cardiomyopathies and cascade screening have been shown to be cost-effective. Early identification of genetic involvement in high-risk family members implies systematic follow-up and for better prognostic outcomes. The implementation of genetic-based medicine in a multidisciplinary approach of heritable CMs will hopefully shift the paradigm from a disease-based treatment to a preventive and individualized medical management.

## Abbreviations

CMs	Cardiomyopathies
ACC	American College of Cardiology
ACM	Arrhythmogenic cardiomyopathy
AHA	American Heart Association
AL	Light chain amyloidosis
ASD	Atrial septal defect
ATPase	Adenozin triphosphate-ase
ATTR	Transthyretin amiloidosis
ATTR-ACT	Safety and Efficacy of Tafamidis in Patients With Transthyretin Cardiomyopathy
ATTRwt	Transthyretin amyloidosis wild-type
AVC	Arrhythmogenic ventricular cardiomyopathy
CHD	Congenital heart disease
CMs	Cardiomyopathies
CWD	Continuous Wave Doppler
DCM	Dilated cardiomyopathy
DNA	Deoxyribonucleic acid
ECG	Electrocardiogram
EMF	Endomyocardial fibrosis
GLS	Global Longitudinal strain
HCM	Hypertrophic cardiomyopathy
HLHS	Hypoplastic left heart syndrome
HOCM	Hypertrophic obstructive cardiomyopathy
ISFC	International Society and Federation of Cardiology
LA	Left atrium
LV	Left ventricle
LVNC	Left ventricular noncompaction
MRI	Magnetic resonance imaging
NGS	Next-Generation Sequencing
NYHA	New York Heart Association
PLN	Phospholamban
PS	Pulmonic stenosis
PWD	Pulsed Wave Doppler
RV	Right ventricle
SCD	Sudden cardiac death
VSD	Ventricular septal defect
VUS	Variant of uncertain significance
WHO	World Health Organization

## References

[1] Arbustini E., Narula N., Dec G.W., Reddy K.S., Greenberg B., Kushwaha S., Marwick T., Pinney S., Bellazzi R., Favalli V. (2013). The MOGE(S) classification for a phenotype-genotype nomenclature of cardiomyopathy: Endorsed by the World Heart Federation. J. Am. Coll. Cardiol..

[2] Braunwald E. (2017). Cardiomyopathies: An Overview. Circ. Res..

[3] McKenna W.J., Maron B.J., Thiene G. (2017). Classification, Epidemiology, and Global Burden of Cardiomyopathies. Circ. Res..

[4] Arbelo E., Protonotarios A., Gimeno J.R., Arbustini E., Barriales-Villa R., Basso C., Bezzina C.R., Biagini E., Blom N.A., de Boer R.A. (2023). 2023 ESC Guidelines for the management of cardiomyopathies. Eur. Heart J..

[5] Vogiatzi G., Lazaros G., Oikonomou E., Lazarou E., Vavuranakis E., Tousoulis D. (2022). Role of genetic testing in cardiomyopathies: A primer for cardiologists. World J. Cardiol..

[6] Hershberger R.E., Givertz M.M., Ho C.Y., Judge D.P., Kantor P.F., McBride K.L., Morales A., Taylor M.R.G., Vatta M., Ware S.M. (2018). Genetic evaluation of cardiomyopathy: A clinical practice resource of the American College of Medical Genetics and Genomics (ACMG). Genet. Med..

[7] Lee H.H., Ching C.K. (2019). Practical Aspects in Genetic Testing for Cardiomyopathies and Channelopathies. Clin. Biochem. Rev..

[8] Charron P. (2006). Clinical genetics in cardiology. Heart.

[9] Popa-Fotea N.M., Micheu M.M., Bataila V., Scafa-Udriste A., Dorobantu L., Scarlatescu A.I., Zamfir D., Stoian M., Onciul S., Dorobantu M. (2019). Exploring the Continuum of Hypertrophic Cardiomyopathy—From DNA to Clinical Expression. Medicina.

[10] Hughes S.E., McKenna W.J. (2005). New insights into the pathology of inherited cardiomyopathy. Heart.

[11] Yang J., Chen S., Duan F., Wang X., Zhang X., Lian B., Kou M., Chiang Z., Li Z., Lian Q. (2022). Mitochondrial Cardiomyopathy: Molecular Epidemiology, Diagnosis, Models, and Therapeutic Management. Cells.

[12] Wilson K.D., Shen P., Fung E., Karakikes I., Zhang A., InanlooRahatloo K., Odegaard J., Sallam K., Davis R.W., Lui G.K. (2015). A Rapid, High-Quality, Cost-Effective, Comprehensive and Expandable Targeted Next-Generation Sequencing Assay for Inherited Heart Diseases. Circ. Res..

[13] Burke M.A., Cook S.A., Seidman J.G., Seidman C.E. (2016). Clinical and Mechanistic Insights Into the Genetics of Cardiomyopathy. J. Am. Coll. Cardiol..

[14] Golbus J.R., Puckelwartz M.J., Fahrenbach J.P., Dellefave-Castillo L.M., Wolfgeher D., McNally E.M. (2012). Population-based variation in cardiomyopathy genes. Circ. Cardiovasc. Genet..

[15] Herman D.S., Lam L., Taylor M.R., Wang L., Teekakirikul P., Christodoulou D., Conner L., DePalma S.R., McDonough B., Sparks E. (2012). Truncations of titin causing dilated cardiomyopathy. N. Engl. J. Med..

[16] Morales A., Goehringer J., Sanoudou D. (2023). Evolving cardiovascular genetic counseling needs in the era of precision medicine. Front. Cardiovasc. Med..

[17] Walsh R., Thomson K.L., Ware J.S., Funke B.H., Woodley J., McGuire K.J., Mazzarotto F., Blair E., Seller A., Taylor J.C. (2017). Reassessment of Mendelian gene pathogenicity using 7,855 cardiomyopathy cases and 60,706 reference samples. Genet. Med..

[18] Couto J.F., Martins E. (2023). Recommendations for the Management of Cardiomyopathy Mutation Carriers: Evidence, Doubts, and Intentions. J. Clin. Med..

[19] Bennette C.S., Gallego C.J., Burke W., Jarvik G.P., Veenstra D.L. (2015). The cost-effectiveness of returning incidental findings from next-generation genomic sequencing. Genet. Med..

[20] Elliott P.M., Anastasakis A., Borger M.A., Borggrefe M., Cecchi F., Charron P., Hagege A.A., Lafont A., Limongelli G., Mahrholdt H. (2014). 2014 ESC Guidelines on diagnosis and management of hypertrophic cardiomyopathy: The Task Force for the Diagnosis and Management of Hypertrophic Cardiomyopathy of the European Society of Cardiology (ESC). Eur. Heart J..

[21] Geske J.B., Ommen S.R., Gersh B.J. (2018). Hypertrophic Cardiomyopathy: Clinical Update. JACC Heart Fail..

[22] Semsarian C., Ingles J., Maron M.S., Maron B.J. (2015). New perspectives on the prevalence of hypertrophic cardiomyopathy. J. Am. Coll. Cardiol..

[23] Michels M., Soliman O.I., Phefferkorn J., Hoedemaekers Y.M., Kofflard M.J., Dooijes D., Majoor-Krakauer D., Cate F.J.T. (2009). Disease penetrance and risk stratification for sudden cardiac death in asymptomatic hypertrophic cardiomyopathy mutation carriers. Eur. Heart J..

[24] Pan S., Caleshu C.A., Dunn K.E., Foti M.J., Moran M.K., Soyinka O., Ashley E.A. (2012). Cardiac structural and sarcomere genes associated with cardiomyopathy exhibit marked intolerance of genetic variation. Circ. Cardiovasc. Genet..

[25] Konno T., Chang S., Seidman J.G., Seidman C.E. (2010). Genetics of hypertrophic cardiomyopathy. Curr. Opin. Cardiol..

[26] Alfares A.A., Kelly M.A., McDermott G., Funke B.H., Lebo M.S., Baxter S.B., Shen J., McLaughlin H.M., Clark E.H., Babb L.J. (2015). Results of clinical genetic testing of 2,912 probands with hypertrophic cardiomyopathy: Expanded panels offer limited additional sensitivity. Genet. Med..

[27] Burns C., Bagnall R.D., Lam L., Semsarian C., Ingles J. (2017). Multiple Gene Variants in Hypertrophic Cardiomyopathy in the Era of Next-Generation Sequencing. Circ. Cardiovasc. Genet..

[28] Maurizi N., Michels M., Rowin E.J., Semsarian C., Girolami F., Tomberli B., Cecchi F., Maron M.S., Olivotto I., Maron B.J. (2019). Clinical Course and Significance of Hypertrophic Cardiomyopathy Without Left Ventricular Hypertrophy. Circulation.

[29] Hershberger R.E., Givertz M.M., Ho C.Y., Judge D.P., Kantor P.F., McBride K.L., Morales A., Taylor M.R., Vatta M., Ware S.M. (2018). Genetic Evaluation of Cardiomyopathy—A Heart Failure Society of America Practice Guideline. J. Card. Fail..

[30] Gersh B.J., Maron B.J., Bonow R.O., Dearani J.A., Fifer M.A., Link M.S., Naidu S.S., Nishimura R.A., Ommen S.R., Rakowski H. (2011). 2011 ACCF/AHA Guideline for the Diagnosis and Treatment of Hypertrophic Cardiomyopathy: A report of the American College of Cardiology Foundation/American Heart Association Task Force on Practice Guidelines. Developed in collaboration with the American Association for Thoracic Surgery, American Society of Echocardiography, American Society of Nuclear Cardiology, Heart Failure Society of America, Heart Rhythm Society, Society for Cardiovascular Angiography and Interventions, and Society of Thoracic Surgeons. J. Am. Coll. Cardiol..

[31] Wheeler M.T., Olivotto I., Elliott P.M., Saberi S., Owens A.T., Maurer M.S., Masri A., Sehnert A.J., Edelberg J.M., Chen Y.-M. (2023). Effects of Mavacamten on Measures of Cardiopulmonary Exercise Testing Beyond Peak Oxygen Consumption: A Secondary Analysis of the EXPLORER-HCM Randomized Trial. JAMA Cardiol..

[32] Rehm H.L., Berg J.S., Brooks L.D., Bustamante C.D., Evans J.P., Landrum M.J., Ledbetter D.H., Maglott D.R., Martin C.L., Nussbaum R.L. (2015). ClinGen The Clinical Genome Resource. N. Engl. J. Med..

[33] Rosca C.I., Branea H.S., Sharma A., Nicoras V.A., Borza C., Lighezan D.F., Morariu S.I., Kundnani N.R. (2023). Rhythm Disturbances in Post-Acute COVID-19 Syndrome in Young Men without Pre-Existing Known Cardiovascular Disease&mdash;A Case Series. Biomedicines.

[34] Yancy C.W., Jessup M., Bozkurt B., Butler J., Casey D.E., Drazner M.H., Fonarow G.C., Geraci S.A., Horwich T., Januzzi J.L. (2013). 2013 ACCF/AHA guideline for the management of heart failure: A report of the American College of Cardiology Foundation/American Heart Association Task Force on practice guidelines. Circulation.

[35] McNally E.M., Mestroni L. (2017). Dilated Cardiomyopathy: Genetic Determinants and Mechanisms. Circ. Res..

[36] Haas J., Frese K.S., Peil B., Kloos W., Keller A., Nietsch R., Feng Z., Müller S., Kayvanpour E., Vogel B. (2015). Atlas of the clinical genetics of human dilated cardiomyopathy. Eur. Heart J..

[37] Fatkin D., Huttner I.G., Kovacic J.C., Seidman J.G., Seidman C.E. (2019). Precision Medicine in the Management of Dilated Cardiomyopathy: JACC State-of-the-Art Review. J. Am. Coll. Cardiol..

[38] Jordan E., Peterson L., Ai T., Asatryan B., Bronicki L., Brown E., Celeghin R., Edwards M., Fan J., Ingles J. (2021). Evidence-Based Assessment of Genes in Dilated Cardiomyopathy. Circulation.

[39] Akinrinade O., Ollila L., Vattulainen S., Tallila J., Gentile M., Salmenperä P., Koillinen H., Kaartinen M., Nieminen M.S., Myllykangas S. (2015). Genetics and genotype-phenotype correlations in Finnish patients with dilated cardiomyopathy. Eur. Heart J..

[40] McNally E.M., Golbus J.R., Puckelwartz M.J. (2013). Genetic mutations and mechanisms in dilated cardiomyopathy. J. Clin. Investig..

[41] Nouhravesh N., Ahlberg G., Ghouse J., Andreasen C., Svendsen J.H., Haunsø S., Bundgaard H., Weeke P.E., Olesen M.S. (2016). Analyses of more than 60,000 exomes questions the role of numerous genes previously associated with dilated cardiomyopathy. Mol. Genet. Genom. Med..

[42] Maggi L., Carboni N., Bernasconi P. (2016). Skeletal Muscle Laminopathies: A Review of Clinical and Molecular Features. Cells.

[43] Hasselberg N.E., Edvardsen T., Petri H., Berge K.E., Leren T.P., Bundgaard H., Haugaa K.H. (2014). Risk prediction of ventricular arrhythmias and myocardial function in Lamin A/C mutation positive subjects. Europace.

[44] Pugh T.J., Kelly M.A., Gowrisankar S., Hynes E., Seidman M.A., Baxter S.M., Bowser M., Harrison B., Aaron D., Mahanta L.M. (2014). The landscape of genetic variation in dilated cardiomyopathy as surveyed by clinical DNA sequencing. Genet. Med..

[45] Liu G.S., Morales A., Vafiadaki E., Lam C.K., Cai W.F., Haghighi K., Adly G., Hershberger R.E., Kranias E.G. (2015). A novel human R25C-phospholamban mutation is associated with super-inhibition of calcium cycling and ventricular arrhythmia. Cardiovasc. Res..

[46] McNair W.P., Sinagra G., Taylor M.R., Di Lenarda A., Ferguson D.A., Salcedo E.E., Slavov D., Zhu X., Caldwell J.H., Mestroni L. (2011). SCN5A mutations associate with arrhythmic dilated cardiomyopathy and commonly localize to the voltage-sensing mechanism. J. Am. Coll. Cardiol..

[47] Tayal U., Prasad S., Cook S.A. (2017). Genetics and genomics of dilated cardiomyopathy and systolic heart failure. Genome Med..

[48] Pinto Y.M., Elliott P.M., Arbustini E., Adler Y., Anastasakis A., Böhm M., Duboc D., Gimeno J., De Groote P., Imazio M. (2016). Proposal for a revised definition of dilated cardiomyopathy, hypokinetic non-dilated cardiomyopathy, and its implications for clinical practice: A position statement of the ESC working group on myocardial and pericardial diseases. Eur. Heart J..

[49] Rosca C.I., Sharma A., Nisulescu D.-D., Otiman G., Duda-Seiman D.-M., Morariu S.I., Lighezan D.F., Kundnani N.R. (2023). Prevalence of Cardio-Embolic Brain Complications in Permanent and Paroxysmal Atrial Fibrillation Patients. Healthcare.

[50] Rosca C.I., Lighezan D.F., Nisulescu D.-D., Sharma A., Neagu M.N., Nistor D., Georgescu D., Kundnani N.R. (2023). Metabolic Syndrome: A Strange Companion of Atrial Fibrillation; A Blessing in Disguise from the Neuropsychiatric Point of View. Biomedicines.

[51] Muchtar E., Blauwet L.A., Gertz M.A. (2017). Restrictive Cardiomyopathy: Genetics, Pathogenesis, Clinical Manifestations, Diagnosis, and Therapy. Circ. Res..

[52] Towbin J.A. (2014). Inherited cardiomyopathies. Circ. J..

[53] Caleshu C., Sakhuja R., Nussbaum R.L., Schiller N.B., Ursell P.C., Eng C., De Marco T., McGlothlin D., Burchard E.G., Rame J.E. (2011). Furthering the link between the sarcomere and primary cardiomyopathies: Restrictive cardiomyopathy associated with multiple mutations in genes previously associated with hypertrophic or dilated cardiomyopathy. Am. J. Med. Genet. Part A.

[54] Gallego-Delgado M., Delgado J.F., Brossa-Loidi V., Palomo J., Marzoa-Rivas R., Perez-Villa F., Salazar-Mendiguchía J., Ruiz-Cano M.J., Gonzalez-Lopez E., Padron-Barthe L. (2016). Idiopathic Restrictive Cardiomyopathy Is Primarily a Genetic Disease. J. Am. Coll. Cardiol..

[55] Brodehl A., Pour Hakimi S.A., Stanasiuk C., Ratnavadivel S., Hendig D., Gaertner A., Gerull B., Gummert J., Paluszkiewicz L., Milting H. (2019). Restrictive Cardiomyopathy is Caused by a Novel Homozygous Desmin (DES) Mutation p.Y122H Leading to a Severe Filament Assembly Defect. Genes.

[56] Cimiotti D., Budde H., Hassoun R., Jaquet K. (2021). Genetic Restrictive Cardiomyopathy: Causes and Consequences—An Integrative Approach. Int. J. Mol. Sci..

[57] Su W., van Wijk S.W., Brundel B. (2022). Desmin variants: Trigger for cardiac arrhythmias?. Front. Cell Dev. Biol..

[58] Kittleson M.M., Maurer M.S., Ambardekar A.V., Bullock-Palmer R.P., Chang P.P., Eisen H.J., Nair A.P., Nativi-Nicolau J., Ruberg F.L., On behalf of the American Heart Association Heart Failure and Transplantation Committee of the Council on Clinical Cardiology (2020). Cardiac Amyloidosis: Evolving Diagnosis and Management: A Scientific Statement From the American Heart Association. Circulation.

[59] Saef J., Martyn T., Ives L., Roth L.R., Grodin J.L., Maurer M.S., Hanna M., Tang W.W. (2023). Predictive Modeling to Assess Pretest Probability of Transthyretin Gene Variants Based on Demographic Information. Circ. Heart Fail..

[60] Pietrangelo A. (2015). Genetics, Genetic Testing, and Management of Hemochromatosis: 15 Years Since Hepcidin. Gastroenterology.

[61] Suzuki H., Ota M., Meguro A., Katsuyama Y., Kawagoe T., Ishihara M., Asukata Y., Takeuchi M., Ito N., Shibuya E. (2012). Genetic characterization and susceptibility for sarcoidosis in Japanese patients: Risk factors of BTNL2 gene polymorphisms and HLA class II alleles. Investig. Ophthalmol. Vis. Sci..

[62] Maurer M.S., Schwartz J.H., Gundapaneni B., Elliott P.M., Merlini G., Waddington-Cruz M., Kristen A.V., Grogan M., Witteles R., Damy T. (2018). Tafamidis Treatment for Patients with Transthyretin Amyloid Cardiomyopathy. N. Engl. J. Med..

[63] te Riele A.S., Tandri H., Bluemke D.A. (2014). Arrhythmogenic right ventricular cardiomyopathy (ARVC): Cardiovascular magnetic resonance update. J. Cardiovasc. Magn. Reson..

[64] Graziano F., Zorzi A., Cipriani A., De Lazzari M., Bauce B., Rigato I., Brunetti G., Pilichou K., Basso C., Marra M.P. (2022). The 2020 “Padua Criteria” for Diagnosis and Phenotype Characterization of Arrhythmogenic Cardiomyopathy in Clinical Practice. J. Clin. Med..

[65] Protonotarios N., Tsatsopoulou A. (2004). Naxos disease and Carvajal syndrome: Cardiocutaneous disorders that highlight the pathogenesis and broaden the spectrum of arrhythmogenic right ventricular cardiomyopathy. Cardiovasc. Pathol..

[66] Vatta M., Marcus F., Towbin J.A. (2007). Arrhythmogenic right ventricular cardiomyopathy: A ‘final common pathway’ that defines clinical phenotype. Eur. Heart J..

[67] Groeneweg J.A., Bhonsale A., James C.A., te Riele A.S., Dooijes D., Tichnell C., Murray B., Wiesfeld A.C., Sawant A.C., Kassamali B. (2015). Clinical Presentation, Long-Term Follow-Up, and Outcomes of 1001 Arrhythmogenic Right Ventricular Dysplasia/Cardiomyopathy Patients and Family Members. Circ. Cardiovasc. Genet..

[68] Towbin J.A., McKenna W.J., Abrams D.J., Ackerman M.J., Calkins H., Darrieux F.C.C., Daubert J.P., de Chillou C., DePasquale E.C., Desai M.Y. (2019). 2019 HRS expert consensus statement on evaluation, risk stratification, and management of arrhythmogenic cardiomyopathy. Heart Rhythm.

[69] van Waning J.I., Moesker J., Heijsman D., Boersma E., Majoor-Krakauer D. (2019). Systematic Review of Genotype-Phenotype Correlations in Noncompaction Cardiomyopathy. J. Am. Heart Assoc..

[70] Zhang W., Chen H., Qu X., Chang C.P., Shou W. (2013). Molecular mechanism of ventricular trabeculation/compaction and the pathogenesis of the left ventricular noncompaction cardiomyopathy (LVNC). Am. J. Med. Genet. Part C Semin. Med. Genet..

[71] Captur G., Syrris P., Obianyo C., Limongelli G., Moon J.C. (2015). Formation and Malformation of Cardiac Trabeculae: Biological Basis, Clinical Significance, and Special Yield of Magnetic Resonance Imaging in Assessment. Can. J. Cardiol..

[72] Petersen S.E., Selvanayagam J.B., Wiesmann F., Robson M.D., Francis J.M., Anderson R.H., Watkins H., Neubauer S. (2005). Left ventricular non-compaction: Insights from cardiovascular magnetic resonance imaging. J. Am. Coll. Cardiol..

[73] Towbin J.A. (2010). Left ventricular noncompaction: A new form of heart failure. Heart Fail. Clin..

[74] Teekakirikul P., Kelly M.A., Rehm H.L., Lakdawala N.K., Funke B.H. (2013). Inherited cardiomyopathies: Molecular genetics and clinical genetic testing in the postgenomic era. J. Mol. Diagn..

[75] Gati S., Papadakis M., Papamichael N.D., Zaidi A., Sheikh N., Reed M., Sharma R., Thilaganathan B., Sharma S. (2014). Reversible de novo left ventricular trabeculations in pregnant women: Implications for the diagnosis of left ventricular noncompaction in low-risk populations. Circulation.

[76] de la Chica J.A., Gómez-Talavera S., García-Ruiz J.M., García-Lunar I., Oliva B., Fernández-Alvira J.M., López-Melgar B., Sánchez-González J., de la Pompa J.L., Mendiguren J.M. (2020). Association Between Left Ventricular Noncompaction and Vigorous Physical Activity. J. Am. Coll. Cardiol..

[77] Probst S., Oechslin E., Schuler P., Greutmann M., Boyé P., Knirsch W., Berger F., Thierfelder L., Jenni R., Klaassen S. (2011). Sarcomere gene mutations in isolated left ventricular noncompaction cardiomyopathy do not predict clinical phenotype. Circ. Cardiovasc. Genet..

[78] van Waning J.I., Caliskan K., Michels M., Schinkel A.F.L., Hirsch A., Dalinghaus M., Hoedemaekers Y.M., Wessels M.W., Ijpma A.S., Hofstra R.M. (2019). Cardiac Phenotypes, Genetics, and Risks in Familial Noncompaction Cardiomyopathy. J. Am. Coll. Cardiol..

[79] Cerrone M., Remme C.A., Tadros R., Bezzina C.R., Delmar M. (2019). Beyond the One Gene-One Disease Paradigm: Complex Genetics and Pleiotropy in Inheritable Cardiac Disorders. Circulation.

